# Non-Survivor Ischemic Stroke Patients Maintain High Serum Caspase-Cleaved Cytokeratin-18 Levels

**DOI:** 10.3390/brainsci10030132

**Published:** 2020-02-27

**Authors:** Leonardo Lorente, María M. Martín, Antonia Pérez-Cejas, Agustín F González-Rivero, Rafael Sabatel, Luis Ramos, Mónica Argueso, Jordi Solé-Violán, Juan J. Cáceres, Alejandro Jiménez, Victor García-Marín

**Affiliations:** 1 Intensive Care Unit. Hospital Universitario de Canarias, Ofra s/n. La Laguna, 38320 Santa Cruz de Tenerife, Spain; 2 Intensive Care Unit, Hospital Universitario Nuestra Señora de Candelaria, Crta del Rosario s/n. 38010 Santa Cruz de Tenerife, Spain; mar.martinvelasco@gmail.com; 3 Laboratory Department, Hospital Universitario de Canarias, Ofra, s/n. La Laguna, 38320 Tenerife, Spain; aperezcejas@gmail.com; 4 Laboratory Department, Hospital Universitario de Canarias, Ofra, s/n. La Laguna, 38320 Santa Cruz de Tenerife, Spain; agonriv@hotmail.com; 5 Department of Radiology, Hospital Universitario de Canarias, Ofra, s/n. La Laguna, 38320 Santa Cruz de Tenerife, Spain; rsabatel@gmail.com; 6 Intensive Care Unit, Hospital General La Palma, Buenavista de Arriba s/n, Breña Alta, 38713 La Palma, Spain; lramosgomez@gmail.com; 7 Intensive Care Unit, Hospital Clínico Universitario de Valencia, Avda. Blasco Ibáñez nº17-19, 46004 Valencia, Spain; moni_begasa@hotmail.com; 8 Intensive Care Unit, Hospital Universitario Dr. Negrín. CIBERES, Barranco de la Ballena s/n, 35010 Las Palmas de Gran Canaria, Spain; jsolvio@gobiernodecanarias.org; 9 Intensive Care Unit, Hospital Insular, Plaza Dr. Pasteur s/n, 35016 Las Palmas de Gran Canaria, Spain; juanjose.caceresagra@gobiernodecanarias.org; 10 Research Unit, Hospital Universitario de Canarias, Ofra s/n. La Laguna, 38320 Santa Cruz de Tenerife, Spain; ajimenezsosa@gmail.com; 11 Department of Neurosurgery, Hospital Universitario de Canarias, Ofra, s/n. La Laguna, 38320 Santa Cruz de Tenerife, Spain; vicgarmar666@gmail.com

**Keywords:** CCCK-18, patients, cerebral infarction, prognosis, mortality

## Abstract

Objective: Caspase-cleaved cytokeratin (CCCK)-18 could appear in blood during apoptosis. In two different studies, on day 1 of cerebral infarction and at 72 h of cerebral infarction, respectively, higher circulating CCCK-18 levels were found in non-surviving than in surviving patients. The objective of this study was to analyze the ability of these levels to predict mortality at any time during the first week of cerebral infarction. Methods: Patients with malignant middle cerebral artery infarction (MMCAI) were included and the diagnosis criteria were the presence, observed in a computed tomography, of an acute cerebral infarction in at least 50% of this territory and midline shift, and an acute neurological deterioration with a Glasgow Coma Scale ≤ 8. Serum CCCK-18 levels at days 1, 4 and 8 of MMCAI were determined. Results: Serum concentrations of CCCK-18 at days 1, 4 and 8 of MMCAI were higher in non-surviving (*n* = 34) than in surviving patients (*n* = 34). Serum CCCK-18 concentrations at days 1, 4 and 8 of MMCAI had an area under curve (95% CI) used to predict a 30-day mortality of 0.83 (0.72 –0.91; *p* < 0.001), 0.78 (0.65–0.89; *p* < 0.001) and 0.82 (0.68–0.92; *p* < 0.001). Conclusions: The novel finding is that serum levels of CCCK-18 levels at any time after the first week of MMCAI could help predict 30-day mortality.

## 1. Introduction

Many disabilities and deaths, and hence the consumption of health resources, result from ischemic stroke [1]. Death occurs at 30 days after an ischemic stroke in 13% to 15% of cases [2] and in 70% of cases of severe ischemic stroke [1]. Several factors have been associated with the poor prognosis of ischemic stroke, such as demographic characteristics (age, sex) [2], clinical severity [2] and blood biomarker levels [3,4]. However, the role of blood concentrations as biomarkers related to apoptosis in patients with ischemic stroke has been scarcely explored. Cell death due to cerebral artery obstruction and cerebral apoptosis appears during ischemic stroke [5,6]. Apoptotic changes have been found in human brain samples after ischemic stroke [7,8,9,10,11,12]. 

Cytokeratin (CK)-18 is a protein belonging to the CK family. CK-18 is, generally, present in the intracytoplasmic cytoskeleton of epithelial tissue. CK-18 is cleaved during apoptosis by the action of caspases and appears as caspase-cleaved cytokeratin (CCCK)-18 in blood [13,14]. In addition, CCCK-18 has also been found in brain samples, such as in the glioma of rats [15] and in the pituitary adenomas of patients [16].

Higher circulating levels of CCCK-18 have been found in non-surviving compared to surviving patients suffering from traumatic brain injury [17] or spontaneous cerebral haemorrhage [18,19,20]. Regarding the blood levels of CCCK-18 levels in patients with ischemic stroke, in a previous study our team found higher circulating CCCK-18 levels on the day of cerebral infarction diagnosis in non-surviving than in surviving patients [21]. In another study, in non-surviving ischemic stroke patients compared to surviving patients, higher circulating CCCK-18 levels were found after 72 h of the ischemic stroke, but not at admission [22]. Thus, we think that the determination of blood levels of CCCK-18 in ischemic stroke patients during follow-up is necessary to describe the evolution of those levels in surviving and non-surviving patients and to determine whether these levels could help the clinician to predict the outcome of these patients at any moment. Our study’s hypothesis was that blood levels of CCCK-18 levels during the first week after an ischemic stroke could be consistently higher in non-surviving than in surviving patients and could be used to predict mortality. Therefore, the objectives of this study were to compare serum CCCK-18 levels during the first week of malignant middle cerebral artery infarction (MMCAI) in surviving and non-surviving patients and to analyze the ability of these levels at any time during the first week of cerebral infarction to predict mortality.

## 2. Methods

### 2.1. Design and Patients

This observational and prospective study was carried out with the approval of the Institutional Board of the 6 hospitals that recruited patients and with the written informed consent of a relative of these patients. The 6 Spanish hospitals recruiting patients were: H. Clínico Universitario de Valencia, H. Universitario Dr. Negrín from Las Palmas de Gran Canaria, H. General de La Palma, H. Universitario de Canarias from La Laguna, H. Insular de Las Palmas de Gran Canaria and H. Universitario Nuestra Señora de Candelaria from Santa Cruz de Tenerife. 

Patients with MMCAI were the group observed in this study. The criteria used for the diagnosis of MMCAI were the presence, observed in a computed tomography, of an acute middle cerebral artery infarction with parenchymal hypodensity in at least 50% of this territory and midline shift, and the presence of an acute neurological deterioration consisting of a Glasgow Coma Scale (GCS) [23] ≤ 8. 

Patients with inflammatory or malignant diseases and under 18 years of age, pregnant, with a subarachnoid or intracerebral haemorrhage, or with only relief measures, were excluded.

The patients were recruited during a period of 24 months between the years 2009 and 2012. Previously, serum CCCK-18 concentrations in some of these patients on day 1 of MMCAI were determined by our team [21]. At this time, we determined serum CCCK-18 concentrations at days 4 and 8 of MMCAI.

### 2.2. Recorded Variables 

Age and sex were recorded, as well as any history of chronic renal faillure, arterial hypertension, heart failure, diabetes mellitus, and chronic obstructive pulmonary disease (COPD). We also recorded GCS, Acute Physiology and Chronic Health Evaluation II (APACHE II) score [24], body temperature, bilirubin, lactic acid, creatinine, sodium, glycemia, partial pressure of arterial oxygen (PaO_2_), fraction of inspired oxygen (FI0_2_), leukocytes, fibrinogen, platelets, international normalized ratio (INR), hemoglobin, activated partial thromboplastin time (aPTT), volumen infarction, hemorrhagic transformation, and midline shift. In addition, we recorded the realization of decompressive craniectomy. The prediction of thirty-day mortality was our end-point of the study.

### 2.3. Blood Samples and Determination of Serum CCCK-18 Concentration 

Serum samples were collected at days 1, 4 and 8 of MMCAI and were maintained at −80 °C until CCCK-18 levels were determined. The serum CCCK-18 concentration determinations were performed in the Laboratory of the Hospital Universitario de Canarias (La Laguna, Spain) with a M30 Apoptosense® ELISA kit (PEVIVA AB, Bromma, Sweden). This kit had the following characteristics: <10% of intra-assay and inter-assay coefficient of variation and a 25 U/L detection limit.

### 2.4. Statistical Methods

We used frequencies (and percentages) and a chi-square test to describe and compare categorical variables. We used medians (and percentile 25 and 75) and a Wilcoxon–Mann–Whitney test to describe and compare continuous variables. To determine the serum CCCK-18 level capacity at days 1, 4, and 8 of the MMCAI for predicting 30-day mortality, analyses of receiver operating characteristics (CPD) were performed and the area under curve (AUC) with 95% confidence intervals (CI) was reported. We also reported sensitivity and specificity, positive and negative likelihood ratios, and positive and negative predicted values with 95% CI of serum levels of CCCK-18 levels cut-offs (selected in basis to Youden J index) at days 1, 4 and 8. We constructed Kaplan–Meier curves using serum CCCK-18 levels of 298 U/L at day 1 (a Youden J index was used for this cut-off selection) and 30-day mortality. To determine the association between serum CCCK-18 levels and mortality, a control for GCS, lactic acid and platelet count was carried out using a multiple logistic regression. We tested the association between continuous variables by Spearman´s rank correlation coefficient. We performed a statistical analysis with the programs SPSS 17.0 (SPSS Inc., Chicago, IL, USA), LogXact 4.1 (Cytel Co., Cambridge, MA, USA) and NCSS 2000 (Kaysville, UT, USA). We considered statistically significant *p*-values < 0.05.

## 3. Results 

Surviving patients (*n* = 34) compared to non-survivors (*n* = 34) showed higher GCS and platelet counts, and lower serum CCCK-18 levels (Table 1). Patient groups showed no significant differences in sex, age, APACHE-II, body temperature, COPD, arterial hypertension, chronic renal failure, heart failure, diabetes mellitus, bilirubin, lactic acid, sodium, leukocytes, creatinine, fibrinogen, glycaemia, haemoglobin, PaO_2_/FIO_2_ ratio, INR, aPTT, thrombolysis, haemorrhagic transformation, volume infarction, midline shift or decompressive craniectomy. 

**Figure 1:**
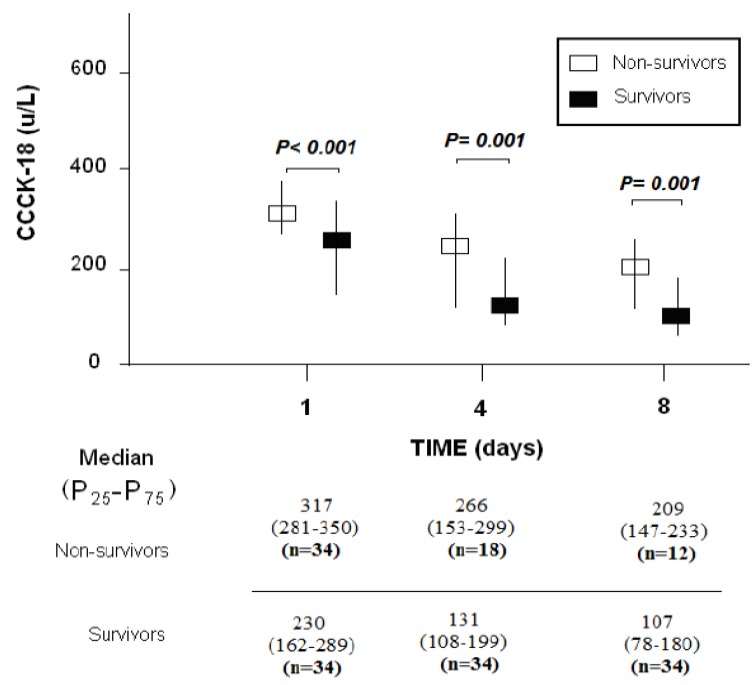
Serum levels of caspase-cleaved cytokeratin (CCCK)-18 levels at days 1, 4 and 8 of MMCAI in 30-day-survivor and non-survivor patients.

In surviving, compared to non-surviving patients, we found lower serum CCCK-18 levels at days 1 (*p* < 0.001), 4 (*p* = 0.001) and 8 (*p* = 0.001) of MMCAI (Figure 1).

**Figure 2:**
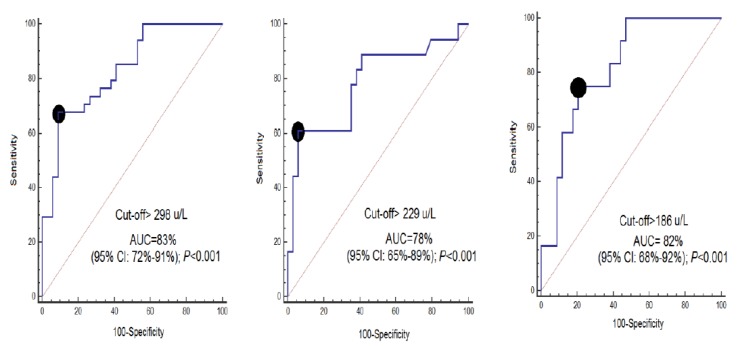
Receiver operation characteristic analysis using serum caspase-cleaved cytokeratin (CCCK)-18 levels at days 1, 4 and 8 of MMCAI as a predictor of mortality at 30 days. AUC, area under curve. CI, confidence intervals.

ROC curve analyses showed that serum CCCK-18 concentrations at days 1, 4, and 8 of MMCAI had an AUC (95% CI) predicting a 30-day mortality of 0.83 (0.72–0.91; *p* < 0.001), 0.78 (0.65–0.89; *p* < 0.001) and 0.82 (0.68–0.92; *p* < 0.001), respectively (Figure 2). Table 2 shows sensitivity and specificity, positive and negative likelihood ratios, and positive and negative predicted values and a 95% CI of serum CCCK-18 levels cut-off at day 1 (>298 U/L), day 4 (>229 U/L) and day 8 (>186 U/L) for mortality prediction.

**Figure 3:**
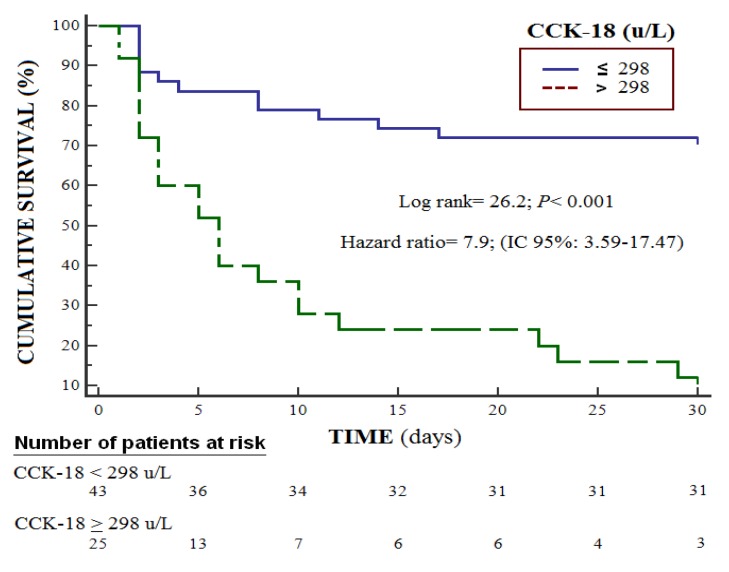
Survival curves at 30 days using serum levels of caspase-cleaved cytokeratin (CCCK)-18 higher or lower than 298 u/L.

Survival analysis showed that patients with serum CCCK-18 levels higher than 298 U/L had a higher risk of death at 30 days (Hazard ratio = 7.9; 95% CI = 3.59–17.47; *p* < 0.001) (Figure 3). Multiple logistic regression showed an association between serum CCCK-18 levels and mortality (OR = 1.023; 95% CI = 1.010–1.037; *p* = 0.001) after consideration of the controls for GCS, lactic acid and platelet count (Table 3).

We found a positive association between the serum levels of CCCK-18 and caspase-3 levels at days 1 (rho = 0.70; *p* < 0.001), 4 (rho = 0.78; *p* < 0.001) and 8 (rho = 0.67; *p* < 0.001) of MMCAI.

## 4. Discussion 

Previously, circulating levels of CCCK-18 were reported in two studies of patients with cerebral infarction [21,22]. Our team had previously determined serum CCCK-levels at day 1 of cerebral infarction and we found higher CCCK-18 levels in non-surviving than in surviving patients [21]. Another study determined plasma CCCK-levels on admission and at 72 h after onset of ischemic stroke. Higher plasma levels of CCCK-18 at 72 h were found in non-surviving than in surviving patients and in patients with worse functional outcome at discharge and at 6 months. However, plasma levels of CCCK-18 on admission were not different between non-surviving and surviving patients and between patients with favorable and unfavorable outcomes [22]. Thus, the novel finding in our study is that serum concentrations of CCCK-18 during the first week of MMCAI were higher in non-surviving patients, which could be used as a predictor of mortality. It is worth noting that higher blood CCCK-18 concentrations at day 1 of cerebral infarction were found in our research but not in the study of Molnar et al. [22]. This could be due to the fact that we only included patients with MMCAI and GCS < 9, however the study of Molnar et al. included patients with ischemic stroke of any cerebral artery territory with any GCS. The 35 surviving patients in the series of Molnar et al. showed 15 points in GCS and the 19 non surviving patients showed a GCS of 10 (95% CI = 5–13). Our patients thus showed a more severe ischemic stroke. We think that our findings with respect to blood CCCK-18 levels at days 1, 4 and 8 of MMCAI could be used for mortality prediction and are interesting because they may be useful to the physician as a biomarker that can help predict the outcome of patients at any time during the first week of MMCAI.

The administration of antiapoptotic agents (ulinastatin, memantine, acetylpuerarin) has reduced neuronal apoptosis and functional neurological damage in ischemic stroke animal models [25,26,27]. Thus, it could be interesting to test the use of antiapoptotic agents in patients with ischemic stroke.

Our study has some limitations, such as that we have not compared CCCK-18 concentrations between serum and plasma, and between MMCAI patients and healthy subjects. Nor have we analyzed CCCK-18 concentrations in brain samples or in cerebrospinal fluid to determine whether an association with serum CCCK-18 levels exists. Serum concentrations of inflammation biomarkers (such as interleukin-6 or tumor necrosis factor-alpha) were also not analyzed to determine their association with serum CCCK-18 levels. In addition, we have not compared the capacity of CCCK-18 to predict mortality with other biomarkers such as S100 calcium-binding protein B (S100B), neuron-specific enolase (NSE), Tau protein, myelin basic protein (MBP), interleukin-6, tumor necrosis factor-alpha, malondialdehyde or matrix metalloproteinase-9 [3,4]. However, we think that a strength of our study is that those higher serum CCCK-18 levels in non-surviving patients were found at the three determinations (days 1, 4 and 8 of MMCAI). Therefore, that those levels at any moment during the first week of a MMCAI, along with other biomarkers, could help clinicians in mortality prediction is the novel and more interesting finding of our study.

## 5. Conclusions

The novel finding is that serum levels of CCCK-18 levels, at any time after the first week of MMCAI, could help predict 30-day mortality.

## Figures and Tables

**Table 1:** Biochemical and clinical characteristics of survivor and non-survivor malignant middle cerebral artery infarction (MMCAI) patients.

	Non-Survivors (*n* = 34)	Survivors (*n* = 34)	*p*-Value
Age (years)—median (*p* 25–75)	63 (53–70)	59 (47–68)	0.36
Female—*n* (%)	13 (38.2)	14 (41.2)	0.99
Heart failure—*n* (%)	1 (2.9)	1 (2.9)	0.99
Diabetes mellitus—*n* (%)	9 (26.5)	4 (11.8)	0.22
COPD—*n* (%)	1 (2.9)	1 (2.9)	0.99
Chronic renal failure—*n* (%)	2 (5.9)	2 (5.9)	0.99
Arterial hypertension—*n* (%)	16 (47.1)	19 (55.9)	0.63
GCS score—median (*p* 25–75)	6 (3–7)	7 (6–8)	0.01
APACHE–II score—median (*p* 25–75)	22 (19–27)	20 (16–25)	0.06
Lactic acid (mmol/L)–median (*p* 25–75)	1.55 (1.00–2.70)	1.20 (0.90–1.70)	0.05
Temperature (°C)—median (*p* 25–75)	36.9 (36.0–37.3)	36.4 (36.0–37.0)	0.15
Bilirubin (mg/dL)—median (*p* 25–75)	0.60 (0.33–1.10)	0.60 (0.40–0.83)	0.95
Glycemia (g/dL)—median (*p* 25–75)	136 (118–162)	127 (100–170)	0.40
Creatinine (mg/dL)—median (*p* 25–75)	1.00 (0.70–1.25)	0.80 (0.60–1.13)	0.19
Sodium (mEq/L)– median (*p* 25–75)	140 (139–145)	139 (136–145)	0.38
PaO2 (mmHg)—median (*p* 25–75)	115 (94–267)	156 (105–293)	0.26
PaO2/FI0_2_ ratio—median (*p* 25–75)	254 (192–325)	300 (198–369)	0.24
INR—median (*p* 25–75)	1.20 (1.01–1.31)	1.06 (1.00–1.20)	0.07
aPTT (seconds)—median (*p* 25–75)	27 (26–32)	28 (25–30)	0.91
Platelets—median × 10^3^/mm^3^ (*p* 25–75)	175 (136–216)	202 (171–265)	0.02
Fibrinogen (mg/dl)—median (*p* 25–75)	419 (337–631)	443 (416–489)	0.90
Leukocytes–median × 10^3^/mm^3^ (*p* 25–75)	13.9 (9.7–20.1)	12.4 (9.6–16.9)	0.32
Hemoglobin (g/dL)—median (*p* 25–75)	12.5 (11.0–14.8)	12.1 (11.4–14.0)	0.81
Thrombolysis—*n* (%)	10 (29.4)	11 (32.4)	0.99
Haemorrhagic transformation—*n* (%)	6 (17.6)	7 (20.6)	0.99
Volumen infarction (mL)—median (*p* 25–75)	180 (60–277)	173 (100–231)	0.64
Midline shift (mm)—median (*p* 25–75)	9.0 (3.5–15.0)	6.0 (2.5–11.5)	0.43
Decompressive craniectomy–*n* (%)	7 (20.6)	9 (26.5)	0.78
CCCK–18 (U/L)—median (*p* 25–75)	317 (281–350)	230 (162–289)	<0.001

*p* 25–75 = percentile 25th–75th^.^; COPD = Chronic Obstructive Pulmonary Disease; GCS = Glasgow Coma Scale; APACHE II = Acute Physiology and Chronic Health Evaluation; PaO_2_ = pressure of arterial oxygen/fraction inspired oxygen; FIO_2_ = pressure of arterial oxygen/fraction inspired oxygen; INR = international normalized ratio; aPTT = activated partial thromboplastin time; CCCK = caspase-cleaved cytokeratin.

**Table 2:** Receiver operation characteristic analyses with serum levels of caspase-cleaved cytokeratin (CCCK)-18 levels at days 1, 4 and 8 of MMCAI for mortality prediction.

	Day 1	Day 4	Day 8
Cut-off of CCCK-18 (U/L)	>298	>229	>186
Specificity and 95% CI	68% (50–83%)	61% (36–83%)	75% (43–95%)
Sensitivity and 95% CI	91% (76–98%)	94% (80–99%)	79% (62–91%)
Positive likelihood ratio and 95% CI	7.7 (2.5–23.2)	10.4 (2.6–41.9)	3.6 (1.7–7.6)
Negative likelihood ratio and 95% CI	0.4 (0.2–0.6)	0.4 (0.2–0.7)	0.3 (0.1–0.9)
Positive predicted value and 95% CI	89% (72–96%)	85% (58–96%)	56% (38–73%)
Negative predicted value and 95% CI	74% (63–82%)	82% (72–89%)	90% (77–96%)

**Table 3:** Multiple logistic regression analysis to predict 30-day mortality.

Variable	Odds Ratio	95% Confidence Interval	*p*
Glasgow Coma Scale (points)	0.749	0.515–1.087	0.13
Lactic acid (mmol/L)	1.050	0.543–2.031	0.89
Platelet count (each 1,000/mm^3^)	0.987	0.976–0.998	0.02
Serum CCCK-18 levels (U/L)	1.025	1.011–1.039	<0.001

CCCK = caspase-cleaved cytokeratin.
